# Threshold adjusted vagus nerve stimulation after asphyxial cardiac arrest results in neuroprotection and improved survival

**DOI:** 10.1186/s42234-022-00092-0

**Published:** 2022-07-20

**Authors:** Rishabh C. Choudhary, Umair Ahmed, Muhammad Shoaib, Eric Alper, Abdul Rehman, Junhwan Kim, Koichiro Shinozaki, Bruce T. Volpe, Sangeeta Chavan, Stavros Zanos, Kevin J. Tracey, Lance B. Becker

**Affiliations:** 1 Laboratory for Critical Care Physiology, Feinstein Institutes for Medical Research, Northwell Health, 350 Community Dr, Manhasset, NY 11030 USA; 2 Institute of Bioelectronic Medicine, Feinstein Institutes for Medical Research, Manhasset, NY USA; 3 Department of Emergency Medicine, Northwell Health, Manhasset, NY USA; 4 Donald and Barbara Zucker School of Medicine at Hofstra/Northwell, Hempstead, NY USA; 5 Zucker School of Medicine at Hofstra/Northwell, Hempstead, NY USA; 6 Center for Molecular Medicine, Feinstein Institutes for Medical Research, Northwell Health, Manhasset, NY USA

**Keywords:** Cardiac arrest, Vagus nerve stimulation, Ischemia-reperfusion injury, Neuroprotection

## Abstract

**Background:**

Vagus nerve stimulation (VNS) has shown therapeutic potential in a variety of different diseases with many ongoing clinical trials. The role of VNS in reducing ischemic injury in the brain requires further evaluation. Cardiac arrest (CA) causes global ischemia and leads to the injury of vital organs, especially the brain. In this study, we investigated the protective effects of customized threshold-adjusted VNS (tVNS) in a rat model of CA and resuscitation.

**Methods:**

Sprague-Dawley rats underwent 12 min asphyxia-CA followed by resuscitation. Rats were assigned to either post-resuscitation tVNS for 2 h or no-tVNS (control). tVNS was applied by electrode placement in the left cervical vagus nerve. To optimize a threshold, we used animal’s heart rate and determined a 15–20% drop from baseline levels as the effective and physiological threshold for each animal. The primary endpoint was 72 h survival; secondary endpoints included neurological functional recovery, reduction in brain cellular injury (histopathology), cardiac and renal injury parameters (troponin I and creatinine levels, respectively).

**Results:**

In comparison to the control group, tVNS significantly improved 72 h survival and brain functional recovery after 12 minutes of CA. The tVNS group demonstrated significantly reduced numbers of damaged neurons in the CA1 hippocampal region of the brain as compared to the control group. Similarly, the tVNS group showed decreased trend in plasma troponin I and creatinine levels as compared to the control group.

**Conclusions:**

Our findings suggest that using tVNS for 2 h after 12 minutes of CA attenuates ischemia neuronal cell death, heart and kidney damage, and improves 72 h survival with improved neurological recovery.

## Background

The therapeutic effect of vagus nerve stimulation (VNS) on neurological recovery (Kim et al., 2019; Sun et al., 2018), regulating metabolic homeostasis, modulating immune function (Borovikova et al., 2000), mitochondrial function (Xue et al., 2017), sepsis (Borovikova et al., 2000; Tracey, 2007), rheumatoid arthritis (Koopman et al., 2016), and hemorrhagic shock (Levy et al., 2013) have been demonstrated in multiple studies. VNS has also been approved for clinical use by the Food and Drug Administration in 1997 and is currently recognized as a safe and effective treatment modality for epilepsy, depression, and stroke (Nemeroff et al., 2006; Koo et al., 2001; Dawson et al., 2021). Furthermore, improved VNS-mediated survival, neuroprotection and improved myocardial function after cardiac arrest have been recently reported (Kim et al., 2019; Sun et al., 2018; Shao et al., 2021).

Cardiac arrest (CA) is the cessation of spontaneous circulation throughout the body following ineffective or absent pumping by the heart. Despite successful resuscitation many survivors still suffer severe neurological injury (Choudhary et al., 2021). CA causes not only ischemic brain injury but also damage to other vital organs. The brain is particularly at risk for ischemic damage due to its high intrinsic metabolic demand and lower intrinsic antioxidant activity (Kristián, 2004; Adibhatla & Hatcher, 2010). Various mechanisms causing cell damage post-CA and resuscitation have been proposed, including cellular damage caused by impairment of cerebral blood flow (Kim et al., 2019; Iordanova et al., 2017), imbalance of the autonomic nervous system resulting in altered vagal activity (Chen et al., 2009), lack of energy production during periods of hypoxia (Kalogeris et al., 2012), and after resuscitation there may be reperfusion injury caused by the release of reactive oxygen species (ROS) (Granger & Kvietys, 2015; Shoaib et al., 2021) and inflammatory cytokines (Jou et al., 2020; Nian et al., 2004). To date, there has been limited success in developing therapeutics that can be used to prevent neuronal damage in the immediate treatment of CA and in addressing the aftermath of reperfusion injury, especially noted in recent failures where no beneficial survival outcomes were observed between targeted hypothermic and normothermic groups for 6 months (Dankiewicz et al., 2021).

Various studies have used different electrical settings to stimulate right or left cervical vagus nerve to titrate benefits. However, the optimal dosage of VNS remains uncertain. For example, Kim et al. used a current strength of 0.05 mA at 1 Hz for 3 h to stimulate left cervical vagus nerve in rats to demonstrate improved cerebral perfusion and neurological outcomes at 24 h after asphyxial CA and ROSC in rats (Kim et al., 2019). By contrast, Sun et al. used 10% reduction in HR as an optimized threshold by stimulating right vagus nerve in rats and demonstrated improved myocardial and cerebral function and longer duration of survival (Sun et al., 2018). Shao et al. recently used a pulse width of 1.2 ms, a frequency of 4 Hz, and an intensity of 6 V to stimulate both right and left vagus nerve in rats (Shao et al., 2021) to produce a survival and neuroprotective effect. Xue et al. used electrical voltage pulses ranging from 2 to 4 V to obtain a 10% reduction in basal heart rate for attenuating isoproterenol-induced cardiac damage in rats (Xue et al., 2017), while Inoue et al. used electrical stimulation (square wave; 50 μA intensity; frequency, 5 Hz; duration, 1 ms) for VNS to mitigate kidney injury following ischemia reperfusion injury in mice (Inoue et al., 2016). All of these studies suggest that VNS gives varied organ protection depending on the stimulation parameters. Thus, the strength, duration of stimulation, time of stimulation, and stimulating electrode placement all play an important role in functional outcomes after VNS and ischemia-reperfusion injury. While previous studies have suggested the benefits of VNS, the effect of adjusting threshold of VNS in-real time in the setting of CA and resuscitation is not well-studied. The current study seeks to evaluate overall survival, cognitive performance, and protective effects of threshold adjusted stimulation of the vagus nerve on the brain, heart, and kidneys in the setting of asphyxial cardiac arrest and resuscitation.

## Material and methods

### Animal experimental protocol for asphyxial-cardiac arrest

**Fig. 1:**
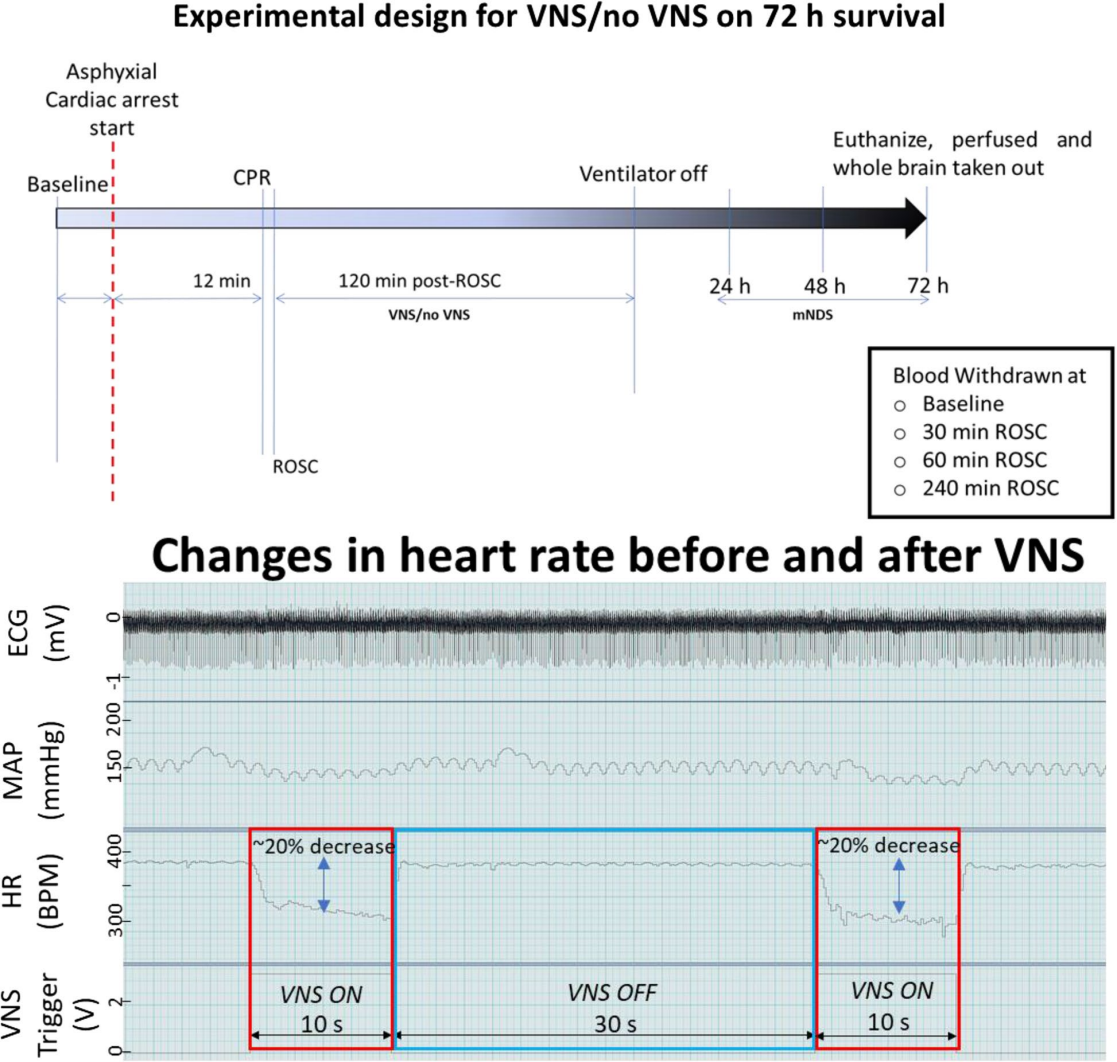
The experimental design for No VNS/VNS after 12 min asphyxial cardiac arrest and resuscitation on 72 h survival (Upper panel). Changes in heart rate before and after VNS indicating 15–20% reduction in basal heart rate as a threshold (Lower panel). (CPR = cardiopulmonary resuscitation; ROSC = return of spontaneous circulation; tVNS = threshold adjusted vagus nerve stimulation; mNDS = modified neurological deficit score

All experiments were performed according to the Institutional Animal Care and Use Committee (IACUC) guidelines at Feinstein Institute for Medical Research (2016–009). Male Sprague-Dawley rats (450–550 g; Charles River Laboratory, Wilmington, MA) were housed on a 12-h light/dark cycle with free access to water and food prior to the experiment. The procedures for inducing asphyxia have been previously published (Han et al., 2010; Shinozaki K et al., 2018). In brief, rats were anesthetized with 4% isoflurane (Isothesia, Butler-Schein AHS), and thereafter, were intubated with a 14-gauge plastic catheter (Surflo, Terumo Medical Corporation, NJ, USA), and placed onto mechanical ventilation with 2% isoflurane. The left femoral artery and vein were cannulated with polyethylene catheters (PE50, BD Intramedic, USA) to measure arterial blood pressure and drug infusion, respectively. An incision of 1–2 cm was made in the neck region to expose left cervical vagus nerve (cVN). After exposure, a cuff electrode was placed around the cVN. After surgical preparation and heparin injection (300 IU) animals were observed until the mean arterial pressure (MAP) was normalized. Baseline recording of mean arterial pressure, heart rate, end-tidal CO2 (ETCO2) were performed, which continued during CA as well as 2 h post return of spontaneous circulation (ROSC) using PowerLab and LabChart (ADInstruments, USA). The procedure for CA began with injecting vecuronium bromide (2 mg/Kg by body weight; Hospira, USA) slowly administered through the left femoral vein over 4 min interval. After 3 min of vecuronium injection, asphyxial-CA was induced by switching off the ventilator and subsequently discontinuing isoflurane. Mean arterial pressure below 20 mmHg was defined as CA (Han et al., 2010). After 12 min of untreated asphyxia-CA, resuscitation was initiated with the resumption of ventilation at 100% oxygen, chest compressions, and a 20 μg/Kg bolus injection of epinephrine (International Medication System, Limited, USA) to achieve ROSC defined as mean arterial pressure (MAP) greater than 60 mmHg. Rats were then monitored on ventilation for 2 h post -ROSC. Esophageal temperature was maintained at 37 ± 0.5 by providing external heat. At 2 h post- ROSC rats were weaned off from the ventilator, all the catheters were removed, and wounds were closed. Rats were then returned to the animal housing facility and provided daily care according to the approved protocol. Animals were monitored for 72 h survival. Animals were then euthanized, and the whole brain was harvested for histological analyses. For comparison with non-CA animals, sham surgery animals were deeply anesthetized and euthanized for tissue collection according to the protocols for the respective experiments. Blood was collected from femoral artery at baseline, ROSC, 30 min, 60 min and 120 min post ROSC for glucose and lactate measurement. A schematic of the experimental protocol is shown in Fig. 1.

### Experimental protocol for threshold adjusted vagus nerve stimulation

Rats were randomly assigned to control (with surgical electrode placement on the vagus nerve but no VNS) or VNS groups. After placing catheters on the femoral artery and vein, the cervical electrode was placed using a midline incision of 1–2 cm in the neck. Salivary glands were separated, and muscles were retracted to visualize the left carotid bundle. The left vagus nerve was carefully isolated from the carotid bundle. A flexible tripolar electrode was then placed on the left cervical vagus nerve (cVN), as used in previous studies (Ahmed et al., 2020; Ahmed et al., 2021). A silicone elastomer was placed on the electrode to prevent current shunting during stimulation (Kwiksil, WPI). In the control group only, electrode was placed on cVN, but stimulation was not delivered. In the tVNS group, immediately after 12 min of CA and ROSC, tVNS was delivered. For the customiized tVNS, the stimulation parameters consisted of symmetrical biphasic rectangular waveforms with a starting current strength of 200 μA, pulse width of 500 μs and frequency of 30 Hz.

tVNS was administered for 10 seconds within a cycle. The duty cycle of our tVNS treatment was 25%, meaning VNS was on 25% of the time but off 75% of the time. A period of the time that takes for one VNS treatment to complete an on-and-off cycle was 40 seconds. Therefore, animals were given 30-second non-VNS time every cycle. The cycle was continued until completing the experiment. We calibrated the tVNS intensity for every tVNS and determined the stimulation thresholds according to the animal’s heart rates during the off time. Heart rates within the last 10 seconds of the off time were averaged and the current strength was increased progressively and incrementally by 20 μA until a 15–20% drop from the averaged value in heart rate (HR) was observed, defined as the threshold which took around ~ 10 min to achieve. This was performed on each animal, the average of all 8 animal thresholds was 360 μA ±45 (Mean ± SEM). Tripolar stimulation with cathode-centered polarity was delivered at the threshold level for 2 hours (10 s ON and 30 s OFF). A STG4008 stimulator was used to deliver tVNS (Multichannel Systems). A schematic of the tVNS and example of the changes in HR is shown in Fig. 1.

### Recording of neurological deficit scores at 24, 48, and 72 h post -resuscitation

Rats that successfully achieved ROSC following 12-min CA were monitored for 72 h survival (*n* = 8 each in VNS and control groups each). To measure neurological recovery a recording of modified Neurological Deficit Score assessment (mNDS) was performed in a blinded manner using a modified version of a previously established method (Neumar et al., 1995). The mNDS values were monitored at 24, 48, and 72 h post- ROSC (mNDS, 0–500; 0, brain dead; 500, normal). mNDS were included to assess general consciousness (100 = conscious; 50 = depressed; 0 = unresponsive), breathing pattern (100 = normal; 50 = moderate; 0 = abnormal), cranial nerve (100 = normal), motor function (50 = normal), sensory (50 = normal), as well as coordination pattern (100 = normal).

### Histology and staining

At 72 h post ROSC, surviving animals were placed under terminal anesthesia and transcardially perfused using cold phosphate buffer saline (PBS, 1X, pH = 7.4; Biorad USA). Sham animals were non-CA rats that were deeply anesthetized and euthanized after perfusion with PBS. Whole brains from sham or experimental groups were harvested and immediately fixed in 4% paraformaldehyde (PFA) at 4 °C. Following cryopreservation in 30% sucrose solution in PBS serial coronal sections (14 μm) containing the hippocampus cornus ammonis 1 (CA1) area were cut on a cryostat (CM1900, Leica) and collected on gelatin subbed glass slides. Nissl staining (Cresyl Violet, Acros Organics, USA), and NeuN staining (Thermofisher Scientific, USA) were performed for neural damage using a previously established method (Jia et al., 2008; Lana et al., 2014), where as Terminal deoxynucleotidyl transferase dUTP nick end labeling (TUNEL) staining (Abcam, UK) was performed as per the manufacturer’s instructions to observe apoptotic cells. For neuronal nuclear protein (NeuN) staining in brief, slides were first incubated with 0.25% Triton X for 20 min (in 1X TBST) followed by blocking with 2% normal donkey serum (NDS; VWR, USA) in 1X, TBST at room temperature. Sections were then incubated with mouse anti-rat NeuN (1:500) antibody prepared in 2% NDS in 1X TBST. Slides were incubated overnight at 4 °C with slight agitation. For double immunostaining, slides were washed 3 times with 1X TBS and incubated with secondary antibody for NeuN (Green, AlexaFluor 488, donkey anti-mouse, 1:400) for 1 h at room temperature. Following incubation, slides were washed three times with 1X TBS followed by water and mounted using Fluoroshield mounting medium with DAPI (Sigma, USA).

After staining sections were imaged at 40X magnification bilaterally using the BX-X800 bright field microscope (Keyence, USA). For nissl, TUNEL, and NeuN staining, we used three serial coronal sections from each group (*n* = 3, sham, control and tVNS). Image J was used to semi-quantify the number of damaged (Nissl staining) and apoptotic cells from CA1 hippocampal and cortex region averaged from both hemispheres. For NeuN, right and left cortex region was imaged using the BX-X800 fluorescent microscope (Keyence, USA) averaged from both hemispheres using image J. All counting was done per focal area.

### Biochemical analysis of troponin 1 and plasma creatinine level after resuscitation

Cardiac and renal injuries were assessed using plasma troponin 1 and creatinine, respectively. Blood plasma from both control and tVNS groups was compared at baseline and at 2 h post-ROSC. Blood samples were withdrawn and then centrifuged at 1000 x g for 10 min to separate the plasma, and the collected plasma was immediately frozen and stored in − 80 °C for further biochemical analysis. Plasma was used to measure Troponin 1 (Abcam, USA) and creatinine (Abcam, USA) levels as per the manufacturer’s protocol.

### Statistical analyses

Data for continuous variables are presented as mean ± standard error of the mean (SEM). For hemodynamic parameter analysis, plasma glucose and lactate measurements, and plasma troponin and creatinine measurements, repeated measures two-way analysis of variance (ANOVA) followed by Sidak’s correction for post-hoc comparison was used to compare differences between groups. The proportion of rats surviving until 72 h was evaluated using the Log-Rank test to compare the survival curves between the two groups. For histologic comparison of brains isolated from sham and the surviving animals from the control and VNS-treated groups, one-way ANOVA followed by Tukey’s multiple comparisons test was used to compare differences among groups. For other analyses, an unpaired two-tailed Student’s t-test was used for continuous variable comparison. Statistical significance was set at *p* < 0.05. GraphPad Prism 9.1 (GraphPad Software Inc., La Jolla, CA, USA) was used for statistical analyses.

## Results

### Vagus nerve stimulation does not alter hemodynamics, plasma glucose or lactate levels after 12 min CA and resuscitation

**Fig. 2:**
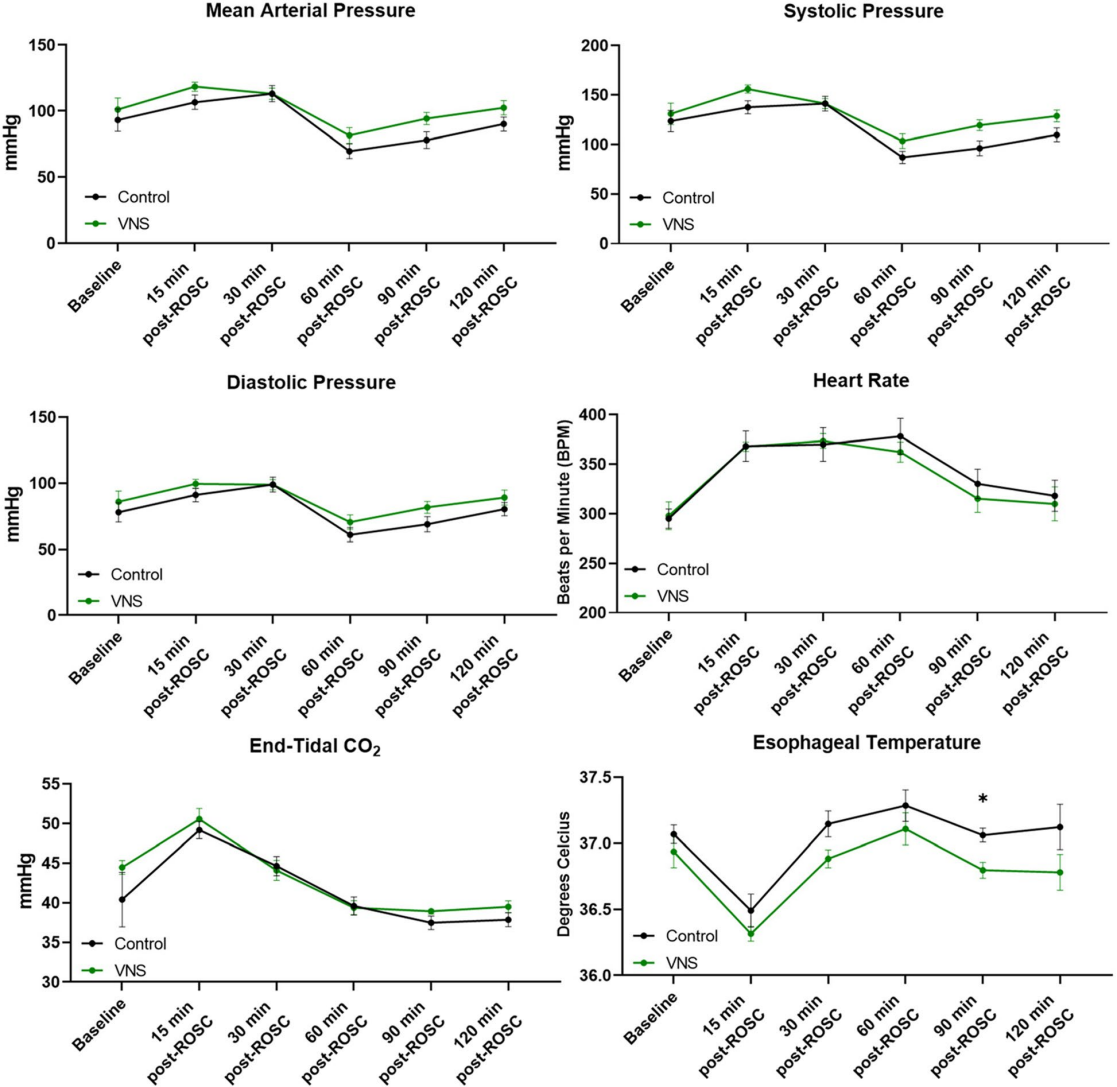
Hemodynamics characteristics. No significant difference was observed between groups. *N* = 8 for control and VNS groups, respectively RM two-way ANOVA with Šídák’s multiple comparisons test for between groups. Only difference between groups was observed in esophageal temperature at 90 min post ROSC

**Fig. 3:**
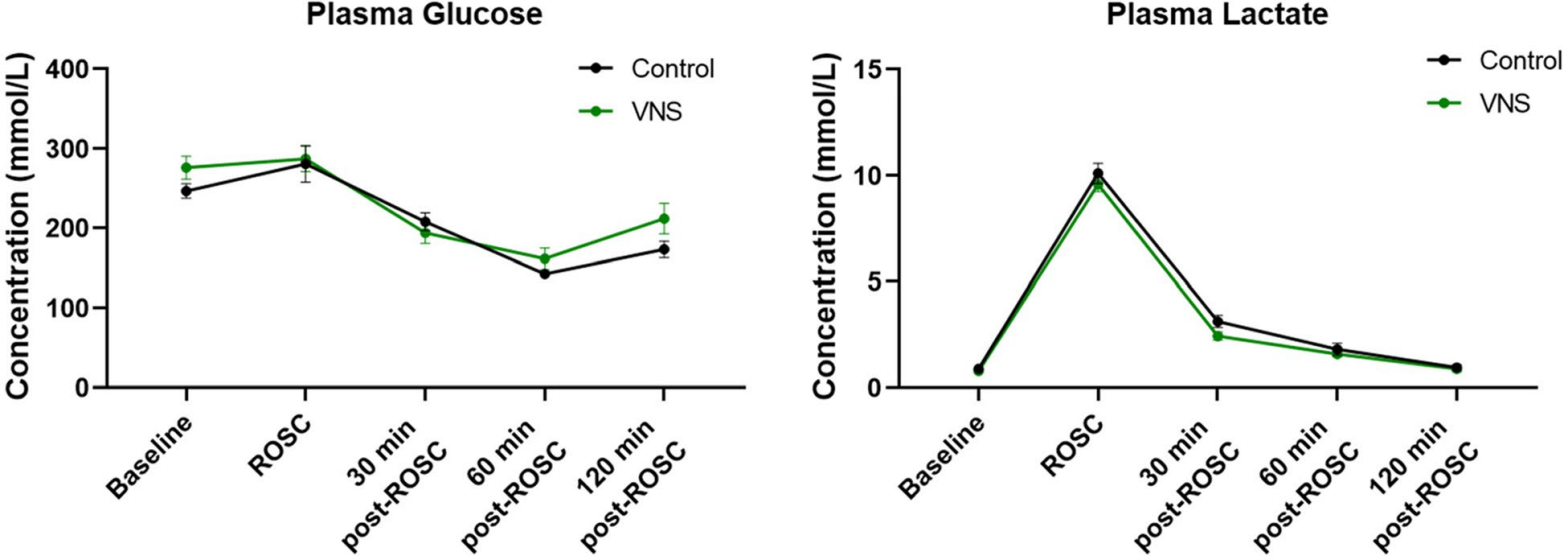
Plasma glucose and lactate level. No significant difference was observed between groups. *N* = 8 for control and VNS groups, respectively RM two-way ANOVA with Šídák’s multiple comparisons test for between groups

The baseline characteristics (body weight), time to achieve CA, or time to achieve ROSC did not significantly differ between control and tVNS-treated rats (Table 1). We used a 15–20% decrease in HR as a threshold to stimulate left cVN after CA and resuscitation. Thus, every animal received an individualized degree of stimulation in terms of strength of the current for tVNS. However, the waveform and duration of stimulation was similar in all tVNS-treated rats. We did not observe changes in hemodynamic parameters, such as MAP, systolic pressure, diastolic pressure, or heart rate in tVNS-treated rats compared to control rats after 12 min CA and resuscitation (Fig. 2). Although lactate levels initially increased post-ROSC as expected, we observed a time-dependent, decreasing trend toward baseline in both groups. Glucose levels tended to decrease up to 60 min post-ROSC and then increase in both groups (Fig. 3). Both glucose and lactate levels were almost identical in both groups post-CA. Taken together, tVNS treatment after CA does not seem to produce any adverse effects on hemodynamics, glucose, and lactate levels.

**Table 1:** Baseline characteristics. No significant difference was observed between groups. *N* = 8 for control and tVNS groups, respectively. Student’s T-test for comparison between means. CA, cardiac arrest; tVNS, threshold adjusted vagus nerve stimulation; ROSC, return of spontaneous circulation. Mean ± SEM

Baseline Characteristics			
	Control (CA - VNS)	Experimental (CA + VNS)	***P*** value
**Weight**	487.3 ± 18.66	490.5 ± 6.083	0.8709
**Time to CA**	181.5 ± 8.464	185.8 ± 9.269	0.7399
**Time to ROSC**	68.13 ± 9.178	54.63 ± 1.580	0.1692

### Vagus nerve stimulation improves survival outcomes and diminishes neurological dysfunction after 12 min CA and resuscitation

**Fig. 4:**
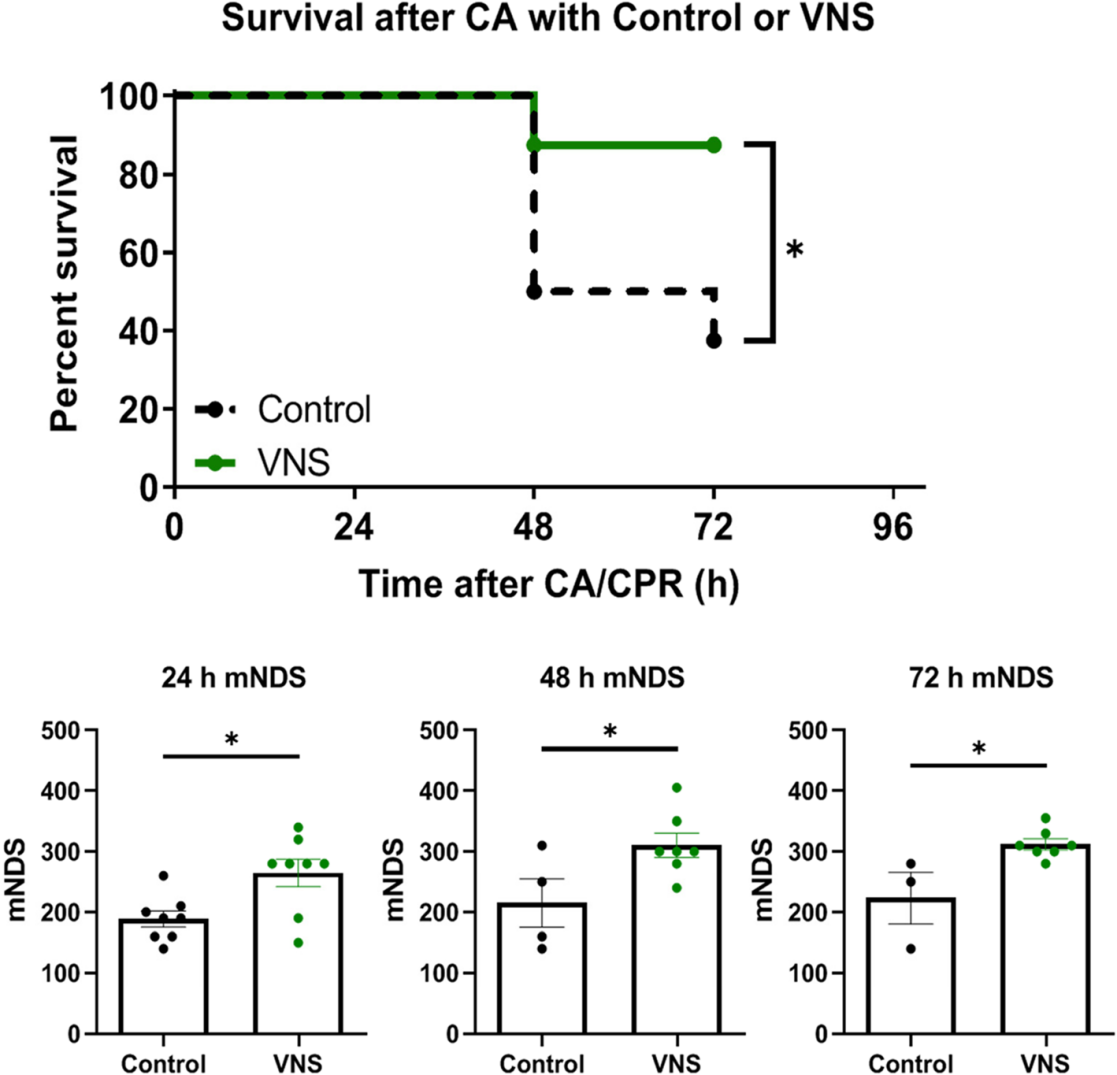
Survival analysis and neurological deficit after CA with and without VNS. A significant survival benefit was observed after CA with the application of tVNS as compared with Control (87.5% v 37.5%, respectively; *P* < 0.05). Evaluation of mNDS at 24, 48, and 72 h post-CA showed a significant decrease in neurological scores in the control group as compared with the VNS group (*P* < 0.05). *N* = 8 each. Log-Rank test was used for survival analysis and Student’s T-test was used for mNDS. tVNS = threshold adjusted vagus nerve stimulation; mNDS = modified neurological deficit score

tVNS animals demonstrates a significantly improved 72 h survival benefit as compared to control rats (87.5% v 37.5%; *P* < 0.05; Fig. 4). Furthermore, in order to assess neurological function, we calculated mNDS at 24, 48, and 72 h after ROSC. The evaluation of mNDS includes general consciousness, breathing pattern, cranial nerve, motor, sensory, and coordination patterns. Results demonstrates that of the surviving rats, tVNS-treated rats have significantly improved neurologic function as compared to vehicle-treated rats (*P* < 0.05 at 24, 48, and 72 h).

### VNS treatment improves brain morphological features and decreases neuronal damage after 12 min CA and resuscitation

**Fig. 5:**
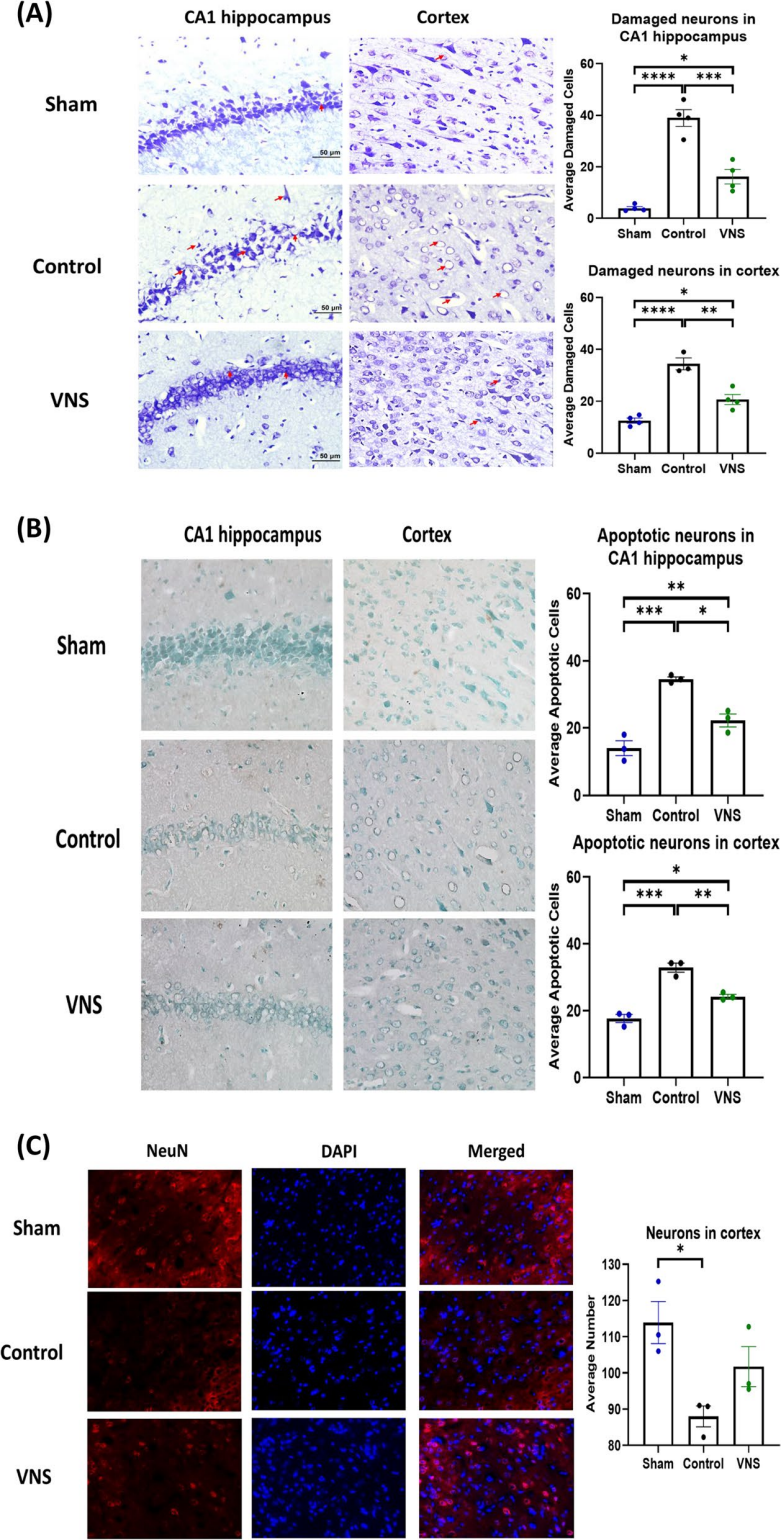
Vagus nerve stimulation decreases neuronal death after 12 min CA and resuscitation. For nissl, TUNEL, and NeuN staining, we used three serial coronal sections from each groups (*n* = 3, sham, control and tVNS). Nissl staining with red arrows indicating damaged neurons in the CA1 of hippocampus and cortex region in brain. The average number of damaged neurons in the CA1 regions of the hippocampus and cortex were significantly increased in CA rats as compared to sham rats (*P* < 0.0001). tVNS administration after CA significantly reduced the number of damaged neurons in CA1 regions of the hippocampus (*P* < 0.001) and cortex (*P* < 0.01) as compared to control (**A**). TUNEL staining shows the apoptotic cells in the CA1 of hippocampus and cortex region in brain. The average number of apoptotic cells in the CA1 regions of the hippocampus were significantly increased in CA rats as compared to sham rats (*P* < 0.001). VNS administration after CA significantly reduced the number of apoptotic cells in CA1 regions of the hippocampus (*P* < 0.01) and cortex (*P* < 0.001) as compared to control (**B**). NeuN staining shows the functional state of neurons. The average number of neurons in the cortex regions of the brain were significantly reduced in CA rats as compared to sham rats (*P* < 0.05) however, no significant differences were observed between sham and VNS groups (**C**). Scale bar 50μm.  Data are presented as mean ± SEM. *P < 0.05, **P < 0.01, ***P < 0.001, and ****P < 0.0001. VNS =  vagus nerve stimulation

Histological analysis of the CA1 hippocampus and cortex region using Nissl and TUNEL staining for the presence of damaged cells and apoptotic cells respectively demonstrates therapeutic benefits of tVNS. A significant increase in the average number of damaged neurons were observed in the CA1 (*P* < 0.0001) and cortex region (*P* < 0.0001) in the control group as compared to sham animals. Although the tVNS group had increased damaged neurons in the CA1 and cortex region as compared with sham (*P* < 0.05), the average number of damaged neurons were significantly reduced after tVNS-treatment as compared with control in the CA1 (*P* < 0.001) and cortex (*P* < 0.01) region respectively (Fig. 5A).

A significant increase in the average number of apoptotic neurons were observed in the CA1 (*P* < 0.001) and cortex region (*P* < 0.001) in the control group as compared to sham animals. Although the tVNS group had increased apoptotic neurons in the CA1 (*P* < 0.01) and cortex (*P* < 0.05) region as compared with sham, the average number of damaged neurons were significantly reduced after tVNS-treatment as compared with control in the CA1 (*P* < 0.05) and cortex (*P* < 0.01) region respectively (Fig. 5B).

We further evaluated the functional states on neurons by NeuN staining in cortex region. A significant decrease in the average number of neurons were observed in the cortex region (*P* < 0.05) in the control group as compared to sham animals while no significant changes in neurons were observed between control and tVNS groups.

Evaluation of the brain cellular features at 72 h post-ROSC shows that ischemia-reperfusion injury is evident in the brain as observed by an increase in the number of damaged neurons and that tVNS-treatment can mitigate the degree of damage as compared to control animals.

### VNS treatment decreases heart and kidney injury after 12 min CA and resuscitation

**Fig. 6:**
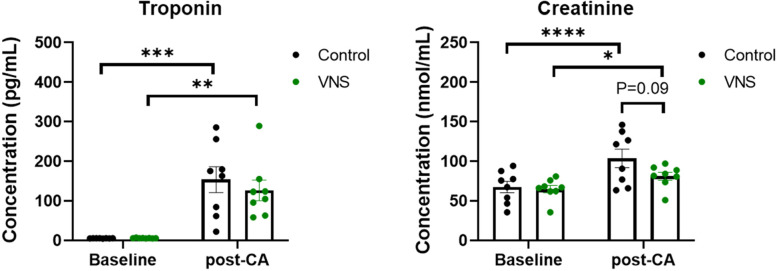
Plasma troponin and creatinine levels after CA with and without VNS. A significant increase in plasma troponin levels was observed in both Control and VNS groups after CA as compared with their respective baseline levels (*P* < 0.001 and *P* < 0.01). However, the increase was less severe after VNS. A significant increase in plasma creatinine levels was observed in both Control and VNS groups after CA as compared with their respective baseline levels (*P* < 0.0001 and *P* < 0.05). The increase in creatine was less severe after tVNS and treatment with tVNS tended to show improvement in creatinine levels as compared with Control (*P* = 0.09). *N* = 8 each. RM two-way ANOVA with Šídák’s multiple comparisons test. tVNS = threshold adjusted vagus nerve stimulation

To assess heart and kidney injury after 12 min CA and resuscitation between tVNS-treated and control rats, we evaluated plasma troponin I and creatinine levels at baseline and at 2 h post-ROSC, respectively. A significant increase in plasma troponin I level was observed in both control and tVNS groups after CA as compared with their respective baseline levels (*P* < 0.001 and *P* < 0.01; Fig. 6). However, the increase was less severe after tVNS treatment suggestive of improvement in heart injury as compared with control.​ Furthermore, a significant increase in plasma creatinine levels was observed in both control and tVNS groups after CA as compared with their respective baseline levels (*P* < 0.0001 and *P* < 0.05). The increase in creatinine was less severe after tVNS, which indicates mitigation of kidney injury as compared with control group (*P* = 0.09). ​These results suggests that VNS-treatment after CA and resuscitation can lead to reduced heart and kidney injury.

## Discussion

In this study we identified that tVNS started immediately after 12 min of CA and resuscitation improves 72 h survival and neurological outcomes at 24, 48 and 72 h after resuscitation. tVNS also attenuates neuronal damage in the hippocampal CA1 and cortex region, reduces neuronal apoptosis, maintain functional state of neurons and reduces heart and kidney injury after CA and resuscitation.

Our results clearly demonstrate that customized left cervical tVNS for 2 h immediately after 12 min CA and resuscitation improves 72 h survival. This result is consistent with a previous study by Sun et al., 2017, who demonstrated that after 8 min of ventricular fibrillation (VF) CA in rats, right cervical VNS initiated for 4 h at the beginning of chest compression with 10% reduction in HR, improved cerebral function, and 72 h survival (Sun et al., 2018). While pre-treatment studies present a range of difficulties, nevertheless, Shao et al. demonstrated that 30 min of VNS to right or left cervical vagus nerve before inducing VF in rats significantly improved 72 h survival (Shao et al., 2021), suggesting that stimulation of either cervical vagus nerve can confer a protective effect. Thus, to activate the array of cervical vagus nerve fibers (afferent and efferent) and achieve optimal effect (Ahmed et al., 2021; Chang et al., 2020), we applied electrical tVNS after 12 min CA and resuscitation, and observed a substantial survival benefit as compared to control (87.5% v 37.5%, respectively; *P* < 0.05; Fig. 4).

In view of our results, consistent with current experiments, it has not escaped our attention that there may be clinical implications. Specifically, our results indicate that customized tVNS improved neurological deficit score at 24, 48, and 72 h, thus tVNS appears to be highly neuroprotective in this model. The clinical scores were further supported by the histological results that demonstrated decreased neuronal injury loss and apoptosis in the hippocampus CA1 and cortex region and also maintaining functional state of neurons for the tVNS treated group. This result is also consistent with previous results that suggested that VNS reduced the cell death at 24 h post-ROSC due to increased cerebral blood flow (Kim et al., 2019), while VNS mediated anti-inflammatory and and anti-apoptotic effect has also been demonstrated in epilepsy (Bie et al., 2021). We further observed an interesting event that out of eight rats which received tVNS, five showed an awakening condition between 25 and 30 min post-ROSC and there were three rats receiving tVNS were able to eat on their own suggesting therapeutic approach of tVNS.

Along with improved brain function, we observed that tVNS also has therapeutic benefits to the heart and kidney. Our results showed that the plasma levels of troponin 1 and creatinine, markers of heart and kidney injury, respectively, were reduced after tVNS administration post-CA and ROSC (Fig. 6). Our result is consistent with a previous study which showed that augmentation of parasympathetic activity by electrical VNS onset at starting of chest compression can achieve hemodynamic improvement through increasing cardiac output after 8 min of VF (Sun et al., 2018). In isoproterenol (ISO)-induced myocardial ischemia in a rat, VNS reduced myocardial infarction, ameliorated cardiomyocyte apoptosis, and cardiac dysfunction (Xue et al., 2017). Recently, in dogs, mild left VNS without changes in HR has been shown to reduce ventricular arrhythmias and improved left ventricular function. This may be attributed to multiple potential mechanisms including reduced cardiac neuronal sprouting, inhibition of excessive sympathetic nerve sprouting, and reduced pro-inflammatory responses (Zhao et al., 2021). The decrease we observed in creatinine levels post-CA with tVNS are in agreement with a prior study which showed that VNS protects against kidney injury induced by ischemia-reperfusion injury through activation of the α7nAChR+ and decreasing pro-inflammatory markers (Inoue et al., 2016). Optogenetic stimulation of the vagus nerve has also been shown to confer kidney protection from ischemia-reperfusion injury in mice (Tanaka et al., 2021). Overall, our findings suggest that in the event of cardiac arrest, tVNS also lowers the risk of heart and kidney injury.

The therapeutic effects of tVNS is likely to be via multifactorial, but in a porcine resuscitation model there are important features of a potential pathophysiological sparing mechanism. For example, VNS partially or completely prevented the development of hyperdynamic circulation, cellular myocardial depression, hyperlactatemia, the shift in the sympathovagal balance toward sympathetic dominance, cardiac mitochondrial dysfunction and reduced the number of activated monocytes (Kohoutova et al., 2019). Also in experiments with myocardial ischemic in rodents, VNS restored mitochondrial dynamics through regulation of Drp1, Fis-1, OPA1 and Mfn1/2; enhanced ATP content and mitochondrial membrane potential; reduced MPTP opening, and improved mitochondrial ultrastructure and size, suggesting that VNS has therapeutic roles in reducing myocardial infarction, ameliorating cardiomyocyte apoptosis, and moderating cardiac dysfunction (Xue et al., 2017). In ischemic stroke patients with atrial fibrillation elevated serum troponin 1 levels were associated with poor neurological outcome (Nam et al., 2020) whereas, in CA patients acute kidney injury was observed and elevated serum creatinine levels resulted in unfavorable neurological outcomes (Hasper et al., 2009). Increased troponin 1 level activates the sympathoadrenal system after ischemic stroke (Barber et al., 2007), sympathoadrenal system activation has been corelated with endothelial damage and poor survival after cardiac arrest in human patients (Johansson et al., 2015). Furthermore, acute kidney injury induced elevated inflammation and altered brain function has also been reported in mice (Liu et al., 2008). These experiments may have implications for our results, as we also observed reduced injury to the heart and kidneys, which could contribute to survival. However, there remains a clear opportunity to further explore and titrate therapeutic modalities for CA and ischemia/reperfusion.

In order to enable more direct clinical translation of our findings, we chose to stimulate the left vagus nerve in our study. Left vagus nerve stimulation (VNS) is an FDA-approved clinical intervention in diseases such as epilepsy and depression (Nemeroff et al., 2006; Koo et al., 2001). The threshold chosen for effective left cervical VNS was a 15–20% reduction of basal HR for stimulating maximal cervical vagus nerve fibers (afferent and efferent) and getting an optimal effect (Ahmed et al., 2021; Chang et al., 2020). Various studies have used different settings to stimulate either right or left cervical vagus nerve for survival and neuroprotection (Kim et al., 2019; Sun et al., 2018; Shao et al., 2021), preventing cardiac damage (Xue et al., 2017) and protecting kidney injury (Inoue et al., 2016). These studies suggest that VNS may have differential effects with different stimulation parameters. In our experimental setup we individually customized different current intensities with a threshold level for achieving 15–20% reduction in HR, thus customized parameters for VNS may be a critical consideration for achieving optimal results.

Our study is not without limitations. During our surgical dissection and placement of electrodes, we may have produced inadvertent direct stimulation of the vagus nerve (Kim et al., 2019). While we attempt to control for this in performing full surgery and electrode placement in the controls, we are not able to discern if electrode placement alone impacted the two groups. In addition, we do not know if our dosage of delivered VNS was a truly optimal dosage. We applied a threshold level of VNS that has been utilized successfully in many prior studies; however, we cannot be certain that this is an optimal dose nor an optimal duration to achieve maximal protection. Furthermore, only male gender rats were utilized in our study and future studies will include both genders. Finally, we note that this is an initial study that is not designed to identify the specific mechanistic pathways that may be responsible for conferring protection. The list of mediators, metabolites, and pathways that have been reported to be altered by VNS is growing and a comprehensive study of VNS mechanism is outside of the scope of the current study; however, this will be a critical area for future studies.

## Conclusion

In this study, we identified that a 15–20% reduction in HR as a threshold to induce vagus nerve stimulation immediately after 12 min CA and resuscitation for 2 h results in significantly improved 72 h survival. tVNS also improves neurological outcomes at 24, 48 and 72 h. tVNS also attenuates neuronal damage in the hippocampal CA1 region and a trend in reduced injury to the heart and kidney after CA and resuscitation.

## Data Availability

All data generated or analyzed during this study are available from the corresponding author upon reasonable request.

## Abbreviations

α7nAChR: alpha 7 nicotinic acetylcholine receptor
CA: Cardiac arrest
cVN: Cervical Vagus nerve
GFAP: Glial fibrillary acidic protein
HR: Heart Rate
MAP: Mean arterial pressure
mNDS: Modified neurological deficit scores
MPTP: Mitochondrial permeability transition pore
NeuN: Neuronal nuclear protein
PBS: Phosphate buffer saline
PFA: Paraformaldehyde
ROS: Reactive oxygen species
ROSC: Return of spontaneous circulation
TBST: (Tris-buffered saline, 0.1% Tween 20)
TUNEL: Terminal deoxynucleotidyl transferase dUTP nick end labeling
tVNS: Threshold-adjusted VNS (tVNS)
VNS: Vagus nerve stimulation
VF: Ventricular fibrillation
